# Effect of neuromuscular reversal with neostigmine/glycopyrrolate versus sugammadex on postoperative ileus following colorectal surgery

**DOI:** 10.1007/s10151-022-02695-w

**Published:** 2022-09-05

**Authors:** L. Traeger, T. D. Hall, S. Bedrikovetski, H. M. Kroon, N. N. Dudi-Venkata, J. W. Moore, T. Sammour

**Affiliations:** 1 Colorectal Unit, Department of Surgery, Royal Adelaide Hospital, Port Road, Adelaide, SA 5000 Australia; 2 Adelaide Medical School, Faculty of Health and Medical Sciences, University of Adelaide, Adelaide, SA Australia; 3 Department of Anaesthesia, Flinders Medical Centre, Bedford Park, SA Australia

**Keywords:** Neostigmine, Glycopyrrolate, Sugammadex, GI-2, Ileus, Colorectal surgery, Acetylcholinesterase inhibitor

## Abstract

**Background:**

Postoperative ileus (POI) is a common complication following colorectal surgery and is mediated in part by the cholinergic anti-inflammatory pathway (CAIP). Neostigmine (acetylcholinesterase inhibitor), co-administered with glycopyrrolate, is frequently given for neuromuscular reversal before tracheal extubation and modulates the CAIP. An alternative reversal agent, sugammadex (selective rocuronium or vecuronium binder), acts independently from the CAIP. The aim of our study was to assess the impact of neuromuscular reversal agents used during anaesthesia on gastrointestinal recovery.

**Methods:**

Three hundred thirty-five patients undergoing elective colorectal surgery at the Royal Adelaide Hospital between January 2019 and December 2021 were retrospectively included. The primary outcome was GI-2, a validated composite measure of time to diet tolerance and passage of stool. Demographics, 30-day complications and length of stay were collected. Univariate and multivariate analyses were performed.

**Results:**

Two hundred twenty-four (66.9%) patients (129 [57.6%] males and 95 [42.4%] females, median age 64 [19–90] years) received neostigmine/glycopyrrolate and 111 (33.1%) received sugammadex (62 [55.9%] males and 49 [44.1%] females, median age 67 [18–94] years). Sugammadex patients achieved GI-2 sooner after surgery (median 3 (0–10) vs. 3 (0–12) days, *p* = 0.036), and reduced time to first stool (median 2 (0–10) vs. 3 (0–12) days, *p* = 0.035). Rates of POI, complications and length of stay were similar. On univariate analysis, POI was associated with smoking history, previous abdominal surgery, colostomy formation, increased opioid use and postoperative hypokalaemia (*p* < 0.05). POI was associated with increased complications, including anastomotic leak and prolonged hospital stay (*p* < 0.001). On multivariate analysis, neostigmine, bowel anastomoses and increased postoperative opioid use (*p* < 0.05) remained predictive of time to GI-2.

**Conclusions:**

Patients who received sugammadex had a reduced time to achieving first stool and GI-2. Neostigmine use, bowel anastomoses and postoperative opioid use were associated with delayed time to achieving GI-2.

**Supplementary Information:**

The online version contains supplementary material available at 10.1007/s10151-022-02695-w.

## Introduction

Postoperative ileus (POI) is a common complication following major abdominal surgery, particularly colorectal surgery, occurring in up to 25% of patients resulting in significant morbidity and mortality [1]. POI occurs in two phases: an initial neurogenic phase followed by a secondary inflammatory phase [1]. The inflammatory phase starts approximately 3 h postoperatively, releasing inflammatory mediators that affect bowel function for a variable length of time [1, 2]. This inflammatory cascade is mediated, in part, by the cholinergic anti-inflammatory pathway (CAIP) [3, 4].

To facilitate abdominal surgery, most patients are paralysed with a non-depolarising neuromuscular blocking drug (NMBD) on induction. These agents competitively antagonise acetylcholine at postsynaptic nicotinic receptors in the neuromuscular junction (NMJ) [5]. Upon completion of surgery, any residual paralysis is reversed before tracheal extubation of the patient with either acetylcholinesterase inhibitors, most commonly neostigmine, or an encapsulating agent named sugammadex. Acetylcholinesterase inhibitors competitively bond with acetylcholinesterase in the synaptic cleft of the NMJ, reducing the hydrolysis of acetylcholine [6]. The increased concentration of acetylcholine competitively reverses the action of the NMBD at the NMJ [7]. The increase in acetylcholine, however, is not limited to the NMJ [8]. Peripheral muscarinic receptors also use acetylcholine and, if left unopposed, produce muscarinic side effects thus require co-administration of an anticholinergic agent (such as glycopyrrolate). The effect of neostigmine and glycopyrrolate as neuromuscular reversal agents on the CAIP and their overall impact on bowel motility following surgery remains unclear [9].

Sugammadex is a modified γ-cyclodextrin that encapsulates the aminosteroid NMBDs, rocuronium and vecuronium, with high affinity [10]. Sugammadex is a large molecule that does not readily enter the NMJ; acting mainly within the circulating plasma. Free NMBD molecules in the plasma are rapidly chelated, creating a concentration gradient promoting the movement of NMBD from the NMJ into the plasma where they are once again sequestered [8]. The reduction in NMBD available at the NMJ, results in the reversal of the neuromuscular blockade. Sugammadex acts independently of cholinergic transmission and therefore does not require co-administration of anticholinergic agents, and thus has no potential to act on the CAIP [11]. Sugammadex is, however, speculated to alter gut motility and gastric emptying due to its affinity to bind with steroid hormones [12, 13].

As sugammadex and neostigmine could influence the return of bowel function, several studies have investigated their impact with varied results [12–16]. However, these studies do not compare neostigmine and sugammadex using a validated gastrointestinal recovery outcome measure, such as GI-2 [17]. Our aim was to identify the effect of neostigmine/glycopyrrolate or sugammadex on gastrointestinal recovery following colorectal surgery using GI-2.

## Materials and methods

This study is reported using the Strengthening the Reporting of Observational studies in Epidemiology (STROBE) guidelines [18], and was approved by the Central Adelaide Local Health Network Human Research Ethics Committee. A waiver of consent for retrospective patients was provided in accordance with the guidelines provided by the National Health and Medical Research Council’s (NHMRC) [19].

### Patient selection

This study was performed at the Colorectal Unit of the Royal Adelaide Hospital (RAH), a tertiary referral centre in South Australia, Australia. Patients were identified from the elective admission lists and underwent surgery between January 2019 and December 2021. All patients at the RAH, are placed on an enhanced recovery pathway (ERP). The ERP protocol can be found at www.tinyurl.com/raheras.

### Inclusion and exclusion criteria

Consecutive elective colorectal patients over 18 years old who underwent major bowel surgery, consisting of large or small bowel resection, reversal or stoma formation, were included. Pelvic exenterations were excluded due to the associated high morbidity and variables affecting return of bowel function. Robotic cases were excluded as they are performed at another geographic site and transferred to the study hospital for postoperative care. Patients who did not receive a neuromuscular reversal agent, received both agents, non-operative admissions, or prescribed acetylcholinesterase inhibitors as part of the ‘Pyridostigmine to reduce the incidence of postoperative ileus following colorectal surgery (PyRICo – P)’ study were excluded [20].

### Data collection

Data were collected retrospectively from paper and electronic medical records by two authors (LT and TH). Anaesthetist choice of neostigmine/glycopyrrolate or sugammadex was collected. Known risk factors for the development of POI were collected [21–23]. Baseline demographics such as age, body mass index (BMI), smoking history, congestive cardiac failure (CCF), chronic obstructive pulmonary disease (COPD), hypertension, diabetes mellitus, regular steroid use, ascites or previous abdominal surgery history were recorded, along with preoperative haemoglobin, total protein and albumin. Operative data included the diagnosis (benign/malignant), surgical approach (open/laparoscopic), laparoscopic to open conversion, procedure type, stoma formation and duration of surgery, and intraoperative and postoperative fluid administration. Postoperative data included opioid requirements in morphine equivalents (intraoperative, postoperative recovery and day one to four use) calculated using Opioid Calculator v2.9.1 (Faculty of Pain Medicine, Australian and New Zealand College of Anaesthetists, Australia),

### Outcomes

The primary outcome was gastrointestinal recovery measured retrospectively using GI-2: a validated outcome measure comprised of time to first stool and tolerance of solid diet without significant nausea or vomiting [17]. Secondary outcomes included POI, defined as not achieving GI-2 by day 4 postoperatively, as well as time to first stool, time to tolerance of oral diet, and nasogastric tube (NGT) reinsertion incidence for both groups. Furthermore, postoperative outcomes including intensive care admission and length of stay were recorded. Thirty-day complications, Clavien-Dindo (CD) grades, return to theatre, and readmission rates were collected [24]. Anastomotic leak was defined by patients having extra-luminal presence of contrast fluid on a contrast-enhanced computed tomography scan and/or evidence of leakage of luminal contents from a surgical join on reintervention within 30 days [25].

### Statistical analysis

A priori power calculation was performed using G*Power 3.1 (Franz Faul, Universitat Kiel, Germany), with the best available data from Hunt et al. showing a mean return of stool with sugammadex of 1.7d (SD 1.2) and 2.2d (SD 1.3) (converted from hours) with neostigmine, as no previous studies used GI-2 [16]. Using an α error of 0.05, ß error of 0.2, power of 0.8 and an effect size of 0.40, a minimum sample size of 100 patients in each arm was required. Numerical data are presented as median (IQR [range]) or mean (standard deviation) depending on parametricity identified with the Shapiro–Wilk test. Univariate analysis was performed using the Mann–Whitney U for nonparametric variables or student's t test for normally distributed continuous variables. The *χ*^2^ or Fisher’s exact test (when expected *n* < 5) for categorical variables. All collected variables were used in the univariate linear regression analysis on log-normal transformed time to GI-2. Statistically significant variables were then used for multivariate linear regression analyses, to determine predictors of GI-2. Data for multivariate linear regression analyses were evaluated and met all linear assumptions. *P* values of < 0.05 were considered statistically significant. A 1-day reduction in GI-2 was considered clinically significant. Statistical analysis was performed using SPSS 28.0 (SPSS Inc., Armonk, NY, USA).

## Results

**Fig. 1:**
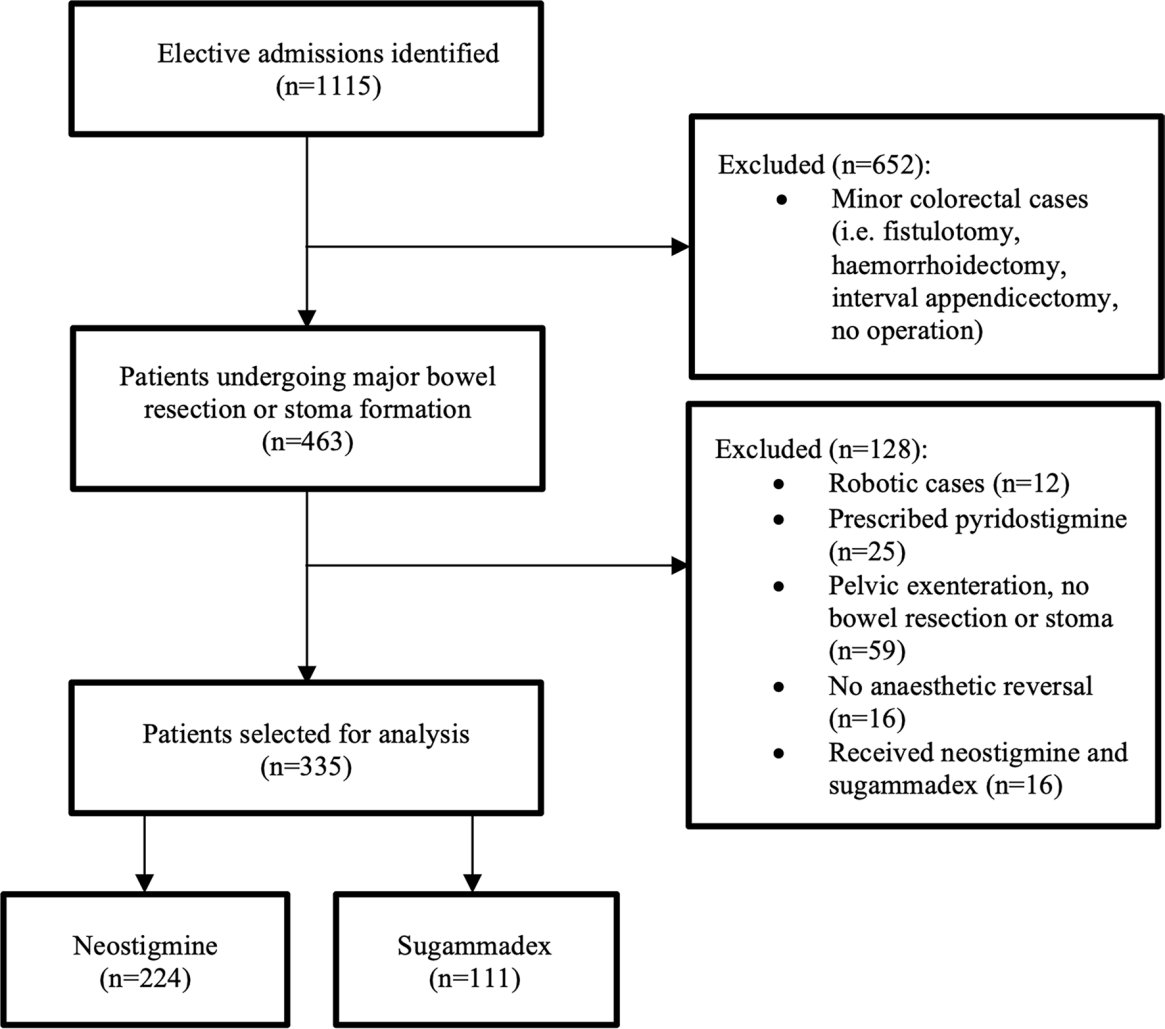
Flowchart of patient selection

Of 1115 elective colorectal admissions during the study period, 335 patients were included (Fig. 1). 224 (66.9%) patients received neostigmine and glycopyrrolate (129 [57.6%] males and 95 [42.4%] females, median age 64 [19–90] years), and 111 (33.1%) received sugammadex (62 [55.9%] males and 49 [44.1%] females, median age 67 [18–94] years). Three patients in the neostigmine group were also given atropine, and seven patients in the sugammadex received glycopyrrolate to treat intraoperative bradycardia. Both groups’ baseline patient and operative characteristics are summarised in Table 1. Patients receiving sugammadex had a higher ASA class > 3 (60.4 vs. 45.1%, *p* < 0.001), a greater BMI (median 28.7 vs. 26.8 kg/m^2^, *p* = 0.003), were more comorbid with COPD (15.3 vs. 6.7%, *p* = 0.012) and hypertension (56.8 vs. 41.5%, *p* = 0.008) and were more likely to undergo laparoscopic surgery (66.7 vs. 50.9%, *p* = 0.006).

**Table 1:** Comparison of baseline patient and operative characteristics between neuromuscular reversal agents*

	Neostigmine (*n* = 224)	Sugammadex (*n* = 111)	*p*-value
Baseline characteristics			
Age; years	64 (53–72 [19–90])	67 (57–76 [18–94])	0.056
Sex			0.763
Female	95 (42.4%)	49 (44.1%)	
Male	129 (57.6%)	62 (55.9%)	
BMI; kg/m^2^	26.8 (23.4–30.4 [15.9 -58.8])	28.7 (24.7–32.9 [18.2 – 73.0])	0.003
ASA			< 0.001
I	5 (2.2%)	3 (2.7%)	
II	118 (52.7%)	41 (36.9%)	
III	101 (45.1%)	62 (55.9%)	
IV	0 (0.0%)	5 (4.5%)	
Smoking history			0.601
Active	46 (20.5%)	19 (17.1%)	
Ex-smoker	66 (29.5%)	38 (34.2%)	
CCF	7 (3.1%)	4 (3.6%)	0.757
COPD	15 (6.7%)	17 (15.3%)	0.012
Hypertension	93 (41.5%)	63 (56.8%)	0.008
Diabetes mellitus			0.074
Prescribed tablets	37 (16.5%)	21 (18.9%)	
Prescribed insulin	2 (0.9%)	5 (4.5%)	
Prescribed regular steroids	9 (4.0%)	10 (9.0%)	0.063
Ascites	2 (0.9%)	4 (3.6%)	0.096
Previous abdominal surgery	135 (60.3%)	59 (53.2%)	0.214
Preoperative haemoglobin; g/L	136 (122–147 [81–177])	134 (121–144 [81–174])	0.221
Preoperative total protein; g/L	73 (70–77 [53–93])	73 (68–78 [56–95])	0.575
Missing	2	2	
Preoperative albumin; g/L	36 (34–40 [20–49])	36 (34–39 [22–46])	0.450
Missing	1	1	
Intraoperative characteristics			
Malignant diagnosis	123 (54.9%)	73 (65.8%)	0.058
Operations			0.888
Right sided ^†^	70 (31.3%)	37 (33.3%)	
Left sided ^‡^	85 (37.9%)	43 (38.7%)	
Total colectomy, pan- proctocolectomy, completion colectomy	16 (7.1%)	10 (9.0%)	
Formation of stoma	8 (3.6%)	3 (2.7%)	
Small bowel resection or ileostomy reversal	45 (20.1%)	18 (16.2%)	
Surgical approach			0.006
Open	110 (49.1%)	37 (33.3%)	
Laparoscopic	114 (50.9%)	74 (66.7%)	
Conversion from laparoscopic to open^§^	19 (16.7%)	16 (21.6%)	0.369
Stoma formed	50 (22.3%)	22 (19.8%)	0.600
Stoma type			0.339
Ileostomy	33 (66.0%)	17 (77.3%)	
Colostomy	17 (34.0%)	5 (22.7%)	
Theatre duration; min	157 (110–194 [42–378])	170 (120–215 [29–433])	0.142
Postoperative characteristics			
Lowest postoperative potassium within POD 1–4, mmol/L	3.8 (3.5–4.0 [2.6–4.8])	3.8 (3.5–4.0 [2.7–5.1])	0.760
Missing	2	0	
Charted aperients	132 (58.9%)	67 (60.4%)	0.802
Intraoperative and recovery opioid use; MEQ	120 (88–163 [20–483])	129 (89–183 [25–768])	0.122
Total opioid use POD 1–4; MEQ	130 (52–227 [0–1831])	135 (57–295 [0–1385])	0.593
Total intraoperative fluids; ml	2000 (1000–2000 [158–5000])	2000 (1000–2000 [100–5000])	0.220
Total recovery fluids; ml	900 (500–1325 [0–3000])	1050 (500–1275 [0–4000])	0.478

*ASA *American society of anaesthesiologists physical status, *BMI* body mass index, *CCF* congestive cardiac failure, *COPD* chronic obstructive pulmonary disease, *MEQ* morphine equivalents, *POD* postoperative day, *POI* postoperative ileus

*Values are median (IQR [range]), mean (SD) or number (percentage)

^†^Includes ileocolic resection, extended/right hemicolectomy, transverse colectomy, subtotal colectomy

^‡^Includes left hemicolectomy, sigmoidectomy, anterior resection, abdominoperineal resection, reversal of Hartmann’s procedure

^§^*n* = 114 neostigmine, *n* = 74 sugammadex

Postoperatively, patients receiving sugammadex had a statistically significantly shortened median time to GI-2 (3 (0–10) vs. 3 (0–12) days, *p* = 0.036) and a reduced median time to first stool (2 (0–10) vs. 3 (0–12), *p* = 0.035) (Table 2). There were no significant differences in time to POI rates, NGT reinsertion, length of stay and 30-day complications between groups (Table 2).

**Table 2:** Postoperative outcomes comparing neuromuscular reversal agents*

	Neostigmine (*n* = 224)	Sugammadex (*n* = 111)	*p*-value
Gastrointestinal recovery			
GI-2; d	3 (2–5 [0–12])	3 (2–4 [0–10])	0.036
Time to first stool; d	3 (2–4 [0–12])	2 (1–4 [0–10])	0.035
Time to tolerance of oral diet; d	2 (1–4 [0–11])	2 (1–4 [0–10])	0.117
POI	65 (29.0%)	28 (25.2%)	0.466
NGT reinsertion	60 (26.8%)	29 (26.1%)	0.898
Complications and clinical outcomes			
ICU admission	11 (4.9%)	3 (2.7%)	0.402
Anastomotic leak^†^	13 (6.7%)	3 (3.0%)	0.279
CD grade			0.830
No complication	97 (43.3%)	43 (38.7%)	
1	22 (9.8%)	11 (9.9%)	
2	86 (38.4%)	50 (45.0%)	
3	8 (3.6%)	3 (2.7%)	
4	11 (4.9%)	4 (3.6%)	
Blood products transfusion required	9 (4.0%)	4 (3.6%)	> 0.999
Return to theatre within 30 days	10 (4.5%)	4 (3.6%)	> 0.999
Readmission within 30 days	28 (12.5%)	13 (11.7%)	0.836
Length of stay; days	5 (4–8 [1–60])	6 (4–8 [2–24])	0.844

*CD* Clavien-Dindo grade, *ICU* intensive care unit, *NGT* nasogastric tube, *POI* postoperative ileus

*Values are median (IQR [range]), mean (SD) or number (proportion)

^†^*n* = 195 for neostigmine, *n* = 99 for sugammadex

Overall, 93 patients (27.8%) had a POI (Table 3). POI was more likely to occur in patients with a history of smoking (62.3 vs. 45.9%, *p *= 0.025), previous abdominal surgery (68.8 vs. 53.7%, *p* = 0.012), those who underwent open surgery (55.9 vs. 39.3%, *p* = 0.006), and patients who had a colostomy formed (60.0 vs. 22.8%, *p* = 0.005). Patients within postoperative day 1–4 with lower potassium (median 3.7 vs. 3.8 mmol/L, *p* = 0.017), charted aperients (69.9 vs. 55.4%, *p* = 0.015) and receiving more postoperative opioids (median 218 vs. 110 MEQ, *p* < 0.001) developed POI. POI was associated with significantly more ICU admissions (9.7 vs. 2.1%, *p* = 0.002), anastomotic leaks (13.9 vs. 2.3%, *p* < 0.001), greater incidence of return to theatre (8.6 vs. 2.5%, *p* = 0.012) and a higher CD grade of complications (*p* < 0.001). Patients diagnosed with a POI had a 3-day increase in median length of stay (8 (3–33) vs. 5 (1–60) days, *p* < 0.001).

**Table 3:** Univariate analysis for postoperative ileus of baseline, intra- and postoperative characteristics, and outcomes*

	Non-POI (*n* = 242)	POI (*n* = 93)	*p*-value
Baseline characteristics			
Age; years	64 (53–73 [18–94])	65 (58–75 [25–89])	0.233
Gender			0.141
Female	110 (45.5%)	34 (36.6%)	
Male	132 (54.5%)	59 (63.4%)	
BMI; kg/m^2^	27.1 (23.8–31.2 [15.9–58.8])	27.3 (24.4–31.6 [15.9 – 73.0])	0.378
ASA class			0.108
I	8 (3.3%)	0 (0.0%)	
II	120 (49.6%)	39 (41.9%)	
III	110 (45.5%)	53 (57.0%)	
IV	4 (1.7%)	1 (1.1%)	
Smoking history			0.025
Active	42 (17.4%)	23 (24.7%)	
Ex-smoker	69 (28.5%)	35 (37.6%)	
CCF	8 (3.3%)	3 (3.2%)	> 0.999
COPD	21 (8.7%)	11 (11.8%)	0.380
Hypertension	111 (45.9%)	45 (48.4%)	0.679
Diabetes mellitus			0.744
Prescribed tablets	43 (17.8%)	15 (16.1%)	
Prescribed insulin	6 (2.5%)	1 (1.1%)	
Prescribed regular steroids	16 (6.6%)	3 (3.2%)	0.298
Ascites	5 (2.1%)	1 (1.1%)	> 0.999
Previous abdominal surgery	130 (53.7%)	64 (68.8%)	0.012
Preoperative haemoglobin; g/L	135 (122–147 [81–177])	134 (122–147 [81–168])	0.786
Preoperative total protein; g/L	73 (69–78 [53–95])	73 (70–76 [58–93])	0.640
Missing	3	1	
Preoperative albumin; g/L	36 (34–40 [22–49])	36 (34–39 [20–49])	0.575
Missing	1	1	
Intraoperative characteristics			
Malignancy diagnosed	146 (60.3%)	50 (53.8%)	0.275
Operation			0.228
Right sided^†^	74 (30.6%)	33 (35.5%)	
Left sided^‡^	88 (36.4%)	40 (43.0%)	
Total colectomy, pan- proctocolectomy, completion colectomy	23 (9.5%)	3 (3.2%)	
Formation of stoma	9 (3.7%)	2 (2.2%)	
Small bowel resection or ileostomy reversal	48 (19.8%)	15 (16.1%)	
Surgical approach			0.006
Open	95 (39.3%)	52 (55.9%)	
Laparoscopic	147 (60.7%)	41 (44.1%)	
Conversion from laparoscopic to open	25 (17.1%)	10 (24.4%)	0.292
Stoma formed	57 (23.6%)	15 (16.1%)	0.138
Stoma type			0.005
Ileostomy	44 (77.3%)	6 (40.0%)	
Colostomy	13 (22.8%)	9 (60.0%)	
Theatre duration; minutes	160 (115–202 [29–433])	161 (118–195 [48–352])	0.969
Postoperative characteristics			
Lowest postoperative potassium within POD 1–4; mmol/L	3.8 (3.6–4.0 [2.6–5.1])	3.7 (3.4–4.0 [2.9–4.6])	0.017
Missing	1	1	
Charted aperients	134 (55.4%)	65 (69.9%)	0.015
Intraoperative and recovery opioid use; MEQ	124 (90–174 [20–768])	120 (80–163 [20–445])	0.571
Total opioid use POD 1–4; MEQ	110 (42–203 [0–1385])	218 (113–439 [10–1831])	< 0.001
Total intraoperative fluids; ml	2000 (1000–2000 [100–5000])	2000 (1000–2000 [158–3000])	0.085
Total recovery fluids; ml	1000 (500–1300 [0–4000])	1000 (500–1400 [0–2500])	0.627
Outcomes			
ICU admission	5 (2.1%)	9 (9.7%)	0.002
Anastomotic leak^§^	5 (2.3%)	11 (13.9%)	< 0.001
Highest CD grade			< 0.001
No complication	140 (57.9%)	0 (0.0%)	
1	33 (13.6%)	0 (0.0%)	
2	59 (24.4%)	77 (82.8%)	
3	5 (2.1%)	6 (6.5%)	
4	5 (2.1%)	10 (10.8%)	
Highest CD grade (excluding POI)			< 0.001
No complication	149 (61.6%)	33 (35.5%)	
1	40 (16.5%)	18 (19.4%)	
2	43 (17.8%)	26 (28.0%)	
3	5 (2.1%)	6 (6.5%)	
4	5 (2.1%)	10 (10.8%)	
Blood products transfusion required	8 (3.3%)	5 (5.4%)	0.380
Return to theatre within 30 days	6 (2.5%)	8 (8.6%)	0.012
Readmission within 30 days	29 (12.0%)	12 (12.9%)	0.818
Length of stay; days	5 (3–6 [1–60])	8 (6–10 [3–33])	< 0.001

*ASA* American society of anesthesiologists physical status, *BMI *body mass index, *CCF* congestive cardiac failure, *CD* Clavien-Dindo grade, *COPD* chronic obstructive pulmonary disease, *ICU* intensive care unit, *MEQ* morphine equivalents, *POD* postoperative day, *POI* postoperative ileus

*Values are median (IQR [range]), mean (SD) or number (proportion)

^†^Includes ileocolic resection, extended/right hemicolectomy, transverse colectomy, subtotal colectomy

^‡^Includes left hemicolectomy, sigmoidectomy, anterior resection, abdominoperineal resection, reversal of Hartmann’s procedure

^§^*n* = 217 for no-POI, *n* = 79 for POI

On univariate and multivariate linear regression analyses, neostigmine/glycopyrrolate use (*p* = 0.034), anastomosis formation (*p* < 0.001) and increased postoperative opioid use were predictive of time to achieving GI-2 (*p* < 0.001) (Table 4).

**Table 4:** Univariate and multivariate linear regression analyses of variables predictive of GI-2

	Univariate			Multivariate		
	*ß*	95% CI	*p*-value	*ß*	95% CI	*p*-value
Neostigmine/Glycopyrrolate use	0.067	(0.008, 0.126)	0.026	0.060	(0.004, 0.116)	0.034
Smoking history	0.058	(0.003, 0.114)	0.041	0.036	(− 0.016, 0.088)	0.175
Previous abdominal surgery	0.057	(0.001, 0.114)	0.047	0.018	(− 0.039, 0.075)	0.543
Open surgical approach	0.081	(0.025, 0.137)	0.005	0.049	(− 0.008, 0.107)	0.093
Anastomosis formed	0.103	(0.035, 0.170)	0.003	0.117	(0.052, 0.181)	< 0.001
Postoperative serum potassium level	0.098	(0.031, 0.166)	0.005	0.064	(0.000, 0.128)	0.051
Charted aperients	0.059	(0.003, 0.116)	0.041	0.053	(0.000, 0.106)	0.051
Postoperative opioids use	0.129	(0.075, 0.184)	< 0.001	0.125	(0.072, 0.179)	< 0.001
Anastomotic leak	0.215	(0.086, 0.344)	0.001	0.082	(− 0.090, 0.254)	0.350
Intensive care unit admission	0.204	(0.065, 0.342)	0.004	0.087	(− 0.053, 0.228)	0.224
Return to theatre	0.187	(0.048, 0.325)	0.008	0.052	(− 0.131, 0.234)	0.578

## Discussion

This study demonstrates a statistically but not clinically relevant difference in time to GI-2 achievement favouring sugammadex used in neuromuscular reversal compared to neostigmine. We also found a clinically significant 1-day reduction in time to first stool favouring sugammadex use. However, the choice of neuromuscular reversal agent did not impact the incidence of POI as defined by GI-2.

These results support previous studies that have demonstrated a reduced time to return of gastrointestinal function with sugammadex. In abdominal surgery studies, sugammadex resulted in an earlier return of flatus when investigating laparoscopic cholecystectomy, but no change in time to first stool [13]. The most extensive study to date included over 8000 patients undergoing abdominal surgery without differentiating types of surgery. It investigated the impact of reversal agents on gastrointestinal recovery, showing that sugammadex resulted in a faster first bowel movement than neostigmine [14]. Several studies have also investigated colorectal surgical patients, favouring sugammadex [15, 16]. In our cohort, although sugammadex patients had an earlier time to first stool, there was no reduction in the risk of developing POI and no clinical difference in time taken for gastrointestinal recovery as defined by GI-2.

Neostigmine did not have a beneficial effect on the return of GI function postoperatively, and there are several plausible explanations for this. The overall duration of action for neostigmine is 20–30 min [26]. Given that the CAIP develops from approximately 3 h postoperatively, this could explain why there is little impact on POI rates. In addition, while historical evidence suggested that co-administration with glycopyrrolate would not reverse the promotility effect of neostigmine [27], contemporary studies have suggested this does lead to a delay in return of gastrointestinal recovery following intraperitoneal surgery [14]. The delay in the return of gastrointestinal function likely results from neostigmine’s cholinergic effects being negated due to its co-administration of the anticholinergic glycopyrrolate. This is supported by the pharmacology of glycopyrrolate, with the duration of action being three to five times longer than neostigmine [28]. This accounts for the observed outcomes of the current study compared to sugammadex, a selective agent without anticholinergic activity [29].

In our study, the reversal agent was chosen by anaesthetist preference, without surgical input. Patients receiving sugammadex were more overweight and comorbid. Compared to neostigmine, sugammadex demonstrates a faster onset of reversal, the potential to reverse deeper neuromuscular blockade, decreased postoperative nausea and vomiting, shortened recovery time, and minimal side effects [30]. Hence, sugammadex was chosen to reverse these higher risk patients to minimise postoperative morbidity. Despite this, the differences in comparing neostigmine/glycopyrrolate and sugammadex, such as BMI and comorbidities, were not identified on multivariate analysis to predict increased GI-2. We, therefore, postulate that these variables do not account for the differences in return of gastrointestinal function.

On multivariate linear regression analysis, bowel anastomoses formation, increased postoperative opioid use and neostigmine use were predictors for a prolonged time to achieving GI-2. Postoperative opioid use has clear associations with delayed return of gastrointestinal function, resulting in increased complications, length of hospital stay and hospital costs [22, 23]. Postoperative opioid use is a modifiable risk factor, with opioid avoidance strategies and interventions such as alvimopan, showing improvements in time to achieve GI-2 [31]. Other studies have also demonstrated, as in our cohort, a link between anastomosis formation and delayed return of bowel function, likely due to increased operative bowel handling [21, 32]. This is also supported by an open surgical approach being associated with delay in return of GI-2, although this did not reach significance on multivariate analyses.

For clinicians, the regular use of sugammadex over neostigmine/glycopyrrolate for neuromuscular reversal is hindered for a few key reasons. During the period of this study, the cost of sugammadex was AU$125 and neostigmine/glycopyrrolate was significantly cheaper at AU$3. The benefits of sugammadex outlined in previous studies and the current study do not outweigh the discrepancy in cost between the two medications [33]. A randomised-blinded study will be required to truly identify the impact sugammadex has on GI-2 and time to first stool. Should this demonstrate a significant clinical improvement in gastrointestinal function recovery, the regular use of sugammadex as part of an ERP could be economically justified, given the financial impact of POI [34]. Furthermore, sugammadex has the potential to cause anaphylaxis [33]. Although this is rare, neostigmine has no risk of anaphylaxis. Given the financial cost of sugammadex and the risk of anaphylaxis, the use of sugammadex for patients remains judicious.

This study had several limitations. This study was retrospective in design. Although there was an attempt to reduce bias using consecutive patients with strict inclusion and exclusion criteria, all selection biases cannot be eliminated. Also, some data points were missing. The baseline characteristics between sugammadex and neostigmine patients differed due to anaesthetist selection based on patient factors. Furthermore a propensity-matched analysis was unable to be performed, as the ratio of the number of relevant predictive variables to the total number of patients in the denominator was too high to present a meaningful analysis. To assess the effects of acetylcholinesterase inhibitors on the development of POI, we are currently recruiting for a double-blinded randomised controlled trial using postoperative acetylcholinesterase inhibitors (pyridostigmine) to investigate this question further (ACTRN:12621000530820).

## Conclusions

This dataset forms the largest cohort of colorectal patients investigating the impact of neostigmine/glycopyrrolate and sugammadex use as neuromuscular reversal agents against the validated outcome of GI-2. Sugammadex use was associated with a shorter time to first stool and GI-2. However, the selection of neuromuscular reversal agents had no significant clinical impact on the development of POI. On multivariate analysis, neostigmine use, bowel anastomoses and increased postoperative opioid use were associated with delayed achievement of GI-2.

## Supplementary Information

Below is the link to the electronic supplementary material.

Supplementary file1 (DOCX 32 KB)

## Data Availability

Not applicable.
